# The emerging roles of N6-methyladenosine (m6A)-modified long non-coding RNAs in human cancers

**DOI:** 10.1038/s41420-022-01050-0

**Published:** 2022-05-09

**Authors:** Jingwen Liu, Wei Zhao, Leyu Zhang, Xi Wang

**Affiliations:** The School and Hospital of Stomatology, Tianjin Medical University, Qixiangtai Road, No. 12, Tianjin, 300070 P.R. China

**Keywords:** Cancer genetics, Gene expression

## Abstract

N^6^-methyladenosine (m^6^A) epitranscriptional modifications widely exist in RNA, which play critical roles in RNA metabolism and biogenesis processes. Long non-coding RNAs (lncRNAs) are class of non-coding RNAs longer than 200 nucleotides without protein-coding ability. LncRNAs participate in a large number of vital biological progressions. With the great improvement of molecular biology, m^6^A and lncRNAs are attracting more attention from researchers and scholars. In this review, we overview the current status of m^6^A and lncRNAs based on the latest research, and propose some viewpoints for future research perspectives.

## FACTS

1. LncRNAs have a series of functions of mediating transcriptional and post-transcriptional gene expression in a wide range of mechanisms and diseases.
2. Biological actions result in the particular transcriptome topology of m^6^A.
3. NcRNAs can feedback to afflict the effect of m^6^A modifications, then complicating the outcomes of pathophysiological activities in vitro and in vivo.

## Background

Given that a wide spectrum of protein-coding RNAs and non-coding RNAs (ncRNAs) can transcribe with the vast majority of genomic sequences, all organisms possess a far more complex transcriptional landscape than we imagined [1–3]. The transcription of ncRNAs is estimated to exceed 90% of the mammalian genome. Recently, evidence has emerged that these non-coding transcripts have biological significance and are indeed not the case of garbage or transcriptional noise [4, 5]. In the extended view of the genome and transcriptome, the current catalog of genetic elements is filled with long non-coding RNAs (lncRNAs) [6]. LncRNAs are ncRNAs with insufficient latency to code protein, which are transcripts of >200 nucleotides in length [7]. Less than 3% of lncRNAs have specified functions. LncRNAs partake in numerous vital biological phenomena, such as enzymatic activity, alternative forms of chromosome conformation, and functional structured RNA domains. Furthermore, unique codes of lncRNA expression function as scaffolds, decoys, or signals to harmonize cell proliferation, differentiation, apoptosis, and metabolism. Little is known about most of the functions of lncRNAs, and even a large quantity of lncRNAs may not have special functions [5, 8, 9].

MicroRNA (miRNAs/miRs), lncRNAs, and circular RNAs (circRNAs) belong to ncRNAs, all of which share a common characteristic that they originate from the transcribed genomes and demonstrate biological functions [10, 11]. CircRNAs are considered to be a category of single-stranded covalently closed RNA molecules that were first discovered in eukaryotes nearly 40 years ago [12]. Compared with lncRNAs, circRNAs can be deemed to a certain extent as a special kind of lncRNAs, which have been modified by an action called back-splicing [13].

N^6^-methyladenosine (m^6^A) epitranscriptional modifications are widely present in RNA, particularly in eukaryotic messenger RNAs (mRNAs), which play pivotal roles in mRNA metabolism and numerous biogenesis processes [8, 14–16]. With new technological advances in RNA modification catalysis, the universal of m^6^A has been published in eukaryotic cells besides its invertibility in mammalian cells [17, 18]. Recently, m^6^A has attracted widespread interest due to its significant influence on cell differentiation, proliferation, migration, invasion, and apoptosis [19, 20]. Three functional proteins, known as “writers”, “readers” and “erasers”, are involved in these internal modifications of m^6^A. All of these internal modifications refer to “epitranscriptomics” [21–23]. These genes that can be affected by m^6^A level changes can exert dramatic effects on cancer progression and growth. This suggests that m^6^A and its regulators may assume a crucial role in the diagnosis and treatment of cancers [7, 22].

Despite some recent substantial progress in the mechanism of m^6^A modifications of ncRNAs, little clarification has been obtained regarding the modification of lncRNAs. In this review, we summarize the role of m^6^A modifications in the mediation and function of lncRNAs and discuss its potential future application and research directions.

## Three kinds of enzymes and detection techniques of m^6^A modifications

The vast bulk of m^6^A modifications result in the unanimity motif of RRm6ACH (in which R represents A or G, and H represents A, C, or U). Accompanied by site and cell/tissue explicitness, m^6^A modifications are generally concentrated in the 3′ untranslated regions (3′-UTRs), that is, they are adjacent to the stop-codons of mRNAs and within long internal exons, contributing to a distinctive m^6^A-derived transcriptome topology [24–26]. Almost all of the features of mRNA processing can be ascribed to the effect of m^6^A functions, like pre-mRNA splicing, export, translation, and stability, therefore exhibiting further influences on the development of humans and diseases [19].

**Fig. 1:**
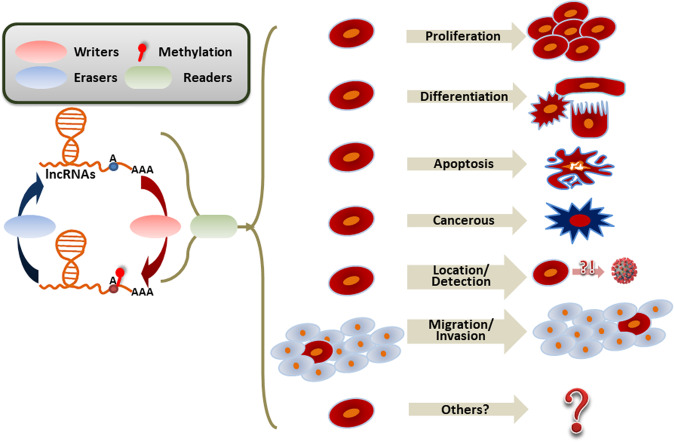
Dynamics and functions of m^6^A processes in lncRNAs. The m^6^A modification procedure is dynamic and invertible. The enzymes in m^6^A modification regulation incorporate “writers” and “erasers”, which respectively assemble and dislodge the methylation and can be identified by “readers”. In biological systems, these effector proteins have substantial signature features which can modify the physiological functions of lncRNAs, including transcription (promote or terminate), expression (advance or silence translation via diverse mechanisms), splicing (affect the performance), stability (enhance the structure or facilitate the degradation), and binding capacity. The entire metabolism can influence the functions of cells.

The m^6^A modification procedure is dynamic and invertible [1]. Enzymes working in the m^6^A modification regulation incorporate “writers” and “erasers” which respectively assemble and dislodge the methylation that can be identified by “readers” [14, 27]. In biological systems, these effector proteins have multiple significant features that can be exhibited by their complicated and changeable functions, which are strongly afflicted by the local environment (Fig. 1).

## Writers

In epitranscriptomics, the writers comprise methyltransferase-like 3/14 (METTL3/14) that can form complexes to ensure the installation of m^6^A methylations into mRNAs [16, 21] and Wilms tumor 1-associating protein (WTAP) that assists METTL3/14 complexes in positioning nuclear spots and ensuring the stability of the complexes [23, 28–30]. The difference between groups of cell lines can indicate the spot effect of METTL3 which is impacted by the protein content proportion of METTL3 and METTL14 and other complexes in adapter subunits. Post-translational modifications (PTMs) proximate to the cytoplasmic presence can show the probability of adjusting the interactions between METTL3 and its partner proteins.

Furthermore, there are other kinds of writers of m^6^A modifications. For instance, Vir-like m^6^A methyltransferase associated (VIRMA, aka KIAA1499) that plays an obvious part in methylation deposition to the 3′-UTR [26], RNA binding motif protein 15/15B (RBM15/15B) that attracts the METTL3/14 complexes to methylate U-riched sites by relying on WTAP [31], and METTL16 that binds to pre-mRNAs and various ncRNAs expand our view of the active m^6^A methyltransferase installed on cellular RNAs [32, 33]. Meanwhile, zinc finger CCCH-type containing 13 (ZC3H13) promotes the “writer” to be located in the nuclear [31]. Recently, METTL5 and ZCCHC4 have been reported to act as modifiers for 18S and 28S ribosomal RNAs (rRNAs), respectively [34, 35]. METTL5 is stabilized by TRMT112, and the action of the METTL5-TRMT112 complex reveals a process very similar to DNA methyltransferases [34, 36]. In the meantime, ZCCHC4 pinpoints to the nucleolus, which assembles the ribosome. The proteins involved in RNA metabolism are overexpressed with the participation of ZCCHC4 [35, 37].

## Erasers

As a reversible chemical process of RNA modifications, erasers are a type of enzyme that can be demethylated during m^6^A modifications. Fat mass and obesity-associated protein (FTO), α-ketoglutarate-dependent dioxygenase homolog 5 (ALKBH5), and ALKBH3 are part of erasers, which significantly influence the biological processes of diseases with a train of complicated interactions that selectively identify m^6^A-labeled target mRNAs [28, 38–40]. So far, FTO and ALKBH5/3 are dominated by a family of α-ketoglutarate-dependent dioxygenases that demethylate m^6^A via Fe^2+^ and α-ketoglutarate [41]. Nevertheless, compared with m^6^A, FTO has been implicated in mRNAs and snRNAs with the demethylation of N^6^ and 2′-O-dimethyladenosine (m^6^A_m_) and in tRNAs with the demethylation of N1-methyladenosine (m^1^A) [42]. Especially in m^6^A_m_ modifications, FTO indicates a tendency to be over-scaled to participate, which can preferentially weaken the stability of mRNAs in cells [43]. Prior research has elucidated that unstable mRNAs may be generated from different sites of FTO in a series of cell lines [38]. ALKBH5 is precisely located in the nucleus. The overexpression of ALKBH5 in cells vastly shrinks the processing of m^6^A in mRNAs [44]. A recent study has revealed that ALKBH3 is inclined to be demethylated on tRNA as opposed to on mRNA or rRNA. Furthermore, ALKBH3 is an undeveloped molecular marker in cancers for its ability as an enzyme to recover DNA damages [45].

## Readers

The ‘readers’ are regarded as a series of proteins that can serve as chemical markers, manipulate RNA structure, or be recruited during the m^6^A modification. There exist several “readers”, like the YT521-B homology (YTH) domain family (including YTHDC1/2 and YTHDF1/2/3) [16, 39, 46], heterogeneous nuclear ribonucleoproteins (including HNRNPA2B1, HNRNPC, and HNRNPG) [47], insulin-like growth factor 2 mRNA binding proteins 1/2/3 (IGF2BP1/2/3) [21], NF-κB-associated protein (NKAP) [48], eukaryotic translation initiation factor 3 (eIF3), proline-rich coiled-coil 2A (Prrc2a), and zinc finger CCCH domain-containing protein 13 (ZC3H13) [49, 50].

The YTH domain has been reported as an m^6^A-binding domain, which was the earliest distinguished reader. YTHDF1 and YTHDF2 give impetus to the translation and decay of m^6^A-methylated mRNAs, respectively. In the cytoplasm, YTHDF3 dramatically increases the metabolism of m^6^A-methylated mRNAs as a group with YTHDF1 and YTHDF2 [46]. YTHDC1 mainly in the nuclear exhibits several functions, such as promoting the export of mRNAs, enhancing the deterioration of specific transcripts, and orchestrating the splicing of mRNAs using the ideal assembly of certain splicing factors [51]. YTHDC2 is related to the stabilization and translation of mRNAs, but the feature of binding domains of RNA remains ambiguous [52]. RNA-protein interactions can be mediated by sites near or around RNA structures that are transformed by m^6^A. Those interactions are called “m^6^A-switch”. HNRNPs, including HNRNPC, HNRNPG, and HNRNPA2B1, modulate selectively tagged transcripts during splicing or processing [28, 53]. Especially with HNRNPA2B1 which mediates the processing of RNAs by m^6^A in the nuclear and peculiarly identifies m^6^A-modified RNAs, eIF3 binds to the 5′-UTR of mRNAs modified by m^6^A to establish the translation expression [30, 54]. Meanwhile, IGF2BPs enhance the stabilization of target genes and related translations [55]. Prrc2a is a novel m^6^A reader, which prefers to bind to a methylated probe, aka the consensus GGACU motif in the coding sequence, to facilitate the stability of mRNA expression [56].

## m^6^A modification detection methods

### Analysis of m^6^A modifications

#### Dot blot

The dot blot (or slot blot) technology, as a study in molecular biology, is a semi-quantitative or quantitative detection of global changes in m^6^A levels in entire or individual RNA species. The dot blot technology remains the favored practice option because of its plain and inexpensive cost. The dot blot technology is simpler to be organized than western blotting, Northern blotting, or Southern blotting but is afflicted by the short susceptibility when low intrinsic specimens were utilized for m^6^A modifications. Nevertheless, with a novel method, Nagarajan et al. [57, 58] generated a technique through an immunoprecipitation-based enrichment of the RNA m^6^A modification step after the dot blot quantification. This technique can be utilized to not only investigate m^6^A modifications of total RNA but also to enrich specific RNAs for the analysis of RNAs in the individual species, such as mRNA, tRNA, rRNA, or miRNA. Nevertheless, regarding m^6^A detection, the dot blot technology can just detect the existence of m^6^A or compare the quantity of m^6^A among various groups [59].

#### The electrochemical immunosensor method

Most analytical techniques present challenges in terms of their achievement and final cost. A novel choice is supplied by the serviceability and susceptibility of the electrochemical immunosensor method that focuses on m^6^A-5′-triphosphate (m^6^ATP), the anti-m^6^A antibody, to track m^6^A. A previous study has manifested the repeatability, specificity, wide linear range, and lack of the detection limit of the immunosensor, which suggests that this method can be the technological basis for RNA and DNA tests for its advantages of being cheaper, convenient, and higher sensitive [60].

### Quantification detection of m^6^A modifications

m^6^A sequencing (m^6^A-seq) and methylated RNA immunoprecipitation sequencing (MeRIP-Seq) are second-generation sequencing after catching m^6^A methylated RNA fragments by m^6^A specific antibodies for the immunoprecipitation technology [20, 27]. m^6^A-level and isoform-characterization sequencing (m^6^A-LAIC-seq) is basically the same as m^6^A-seq but widens the perception of m^6^A biological characteristics and depends on m^6^A-IP of full-length poly (A) ^+^ RNA [61, 62]. However, this method can be applied to measure the levels of m^6^A at each site but not stoichiometrically analyze the single modified nucleotide [7, 61]. All of these three methods are the most common biological techniques which have been adopted in the detection of m^6^A.

Site-specific cleavage and radioactive labeling followed by ligation-assisted extraction and thin-layer chromatography (SCARLET), is an accessible technique to research the biological functions of RNA modifications using reachable equipment and materials, together with 5-methylcytosine, pseudouridine, and 2′-O-methyl ribonucleosides. In m^6^A, SCARLET can be employed to estimate the exact sites and status of m^6^A modifications in any supposed locations of mRNAs/lncRNAs with an individual resolution of nucleotides. It can help m^6^A-seq, MeRIP-Seq, and m^6^A-LAIC-seq to precisely determine the single-base resolution of RNAs [7, 63, 64].

Besides immunoprecipitation enrichment, there are some other methods to detect m^6^A, including crosslinking and immunoprecipitation (CLIP) [65, 66]. The CLIP builds covalent bonds between proteins and RNAs with direct contact with ultraviolet (UV) light irradiation and is a state-of-the-art method that spread the practice to stabilize direct protein-RNA interactions. This technology, initially matured in Escherichia coli, is widely recognized as an essential procedure for researching dynamic protein interactions in multitudes of cellular processes in multifarious biological systems [67, 68]. CLIP consists of photo-crosslinking-assisted m^6^A sequencing (PA-m^6^A-Seq) [69], m^6^A individual-nucleotide-resolution cross-linking and immunoprecipitation (miCLIP) [70], and m^6^A-CLIP. The amplification of polymerase chain reaction (PCR) can advance the comparatively poor efficiency of this reaction, and the extremely purified protein-RNA complexes can potentially be obtained by the covalent binding. Arguello et al. advanced a chemical proteomics approach that depends on photo-cross-linking with synthetic diazirine-containing RNA probes and quantitative proteomics to profile RNA-protein interactions modulated by m^6^A. The proteins that can be used for combination are YTH domain-containing (reader) and ALKBH5 (eraser) [71]. However, the accuracy of the detection is limited by the fact that the crosslink is still feasible to be constructed in adjacent RNA sequences and that UV light has efficient penetrability.

The sequence-specific endoribonuclease MazF belongs to Escherichia coli toxins, which is engaged in growth mediation in response to stress. It includes MAZTER-seq and m^6^A-sensitive RNA-Endoribonuclease-Facilitated sequencing (m^6^A-REF-seq). Interestingly, with the specific capability against ACA sequences, endoribonuclease is sensitive to m^6^A to degrade mRNAs, decay protein synthesis, and enhance growth arrest. Theoretically, in the presence of MazF, every piece should start at the ACA site (5′ACA) and end downstream at the ACA site (3′ACA) [72, 73]. Mounting evidence unravels that the RNA cleavage reaction at the 5′-ACA-3′ site occurs only in single-stranded RNAs and never in double-stranded RNAs [74]. However, little is known about the susceptibility of MazF to the methylated presence of m^6^A bases in structured RNAs.

Deamination-adjacent to RNA modification target sequencing (DART-seq), an antibody-free process to assess m^6^A locations, APOBEC1 expression leads to C-to-U deamination near the sites of m^6^A residues after the binding of the cytidine deaminase APOBEC1 to the YTH domain of m^6^A. It is also possible to isolate thousands of m^6^A sites in 10 ng entire RNA and to identify the quantification of m^6^A aggregation in cells. Furthermore, the long-read DART-seq can harvest the distribution of m^6^A among the individual transcripts. However, the functional quality of DART-seq is currently limited in vitro [75].

High-resolution melting (HRM) analysis is a high-throughput measurement method that can be utilized to check m^6^A modification as an elementary process. The RNA mixed-specimens can be modified stably from 100% methylation to 100% unmethylation with the HRM analysis. The presence of m^6^A can be easily characterized at the special position of RNA using entire RNA samples and qPCR machines by HRM. Although the monomethylation of the adenosine exocyclic amino group cannot change the hydrogen bonding in the nucleic acid duplex, it can influence the stacking interaction that can impact the melting properties. The HRM analysis may deepen the understanding of the dynamic modification of certain RNAs [76].

Recently, two innovative techniques have been developed for RNA modification detection. Wang et al. researched that with the help of dithiothreitol (DTT)-mediated thiol-addition chemical reactions, the unsteady N^6^-hydroxymethyladenosine (hm^6^A) can be transformed into the extra sturdy N^6^-dithiolsitolmethyladenosine (dm^6^A). An FTO-assisted m^6^A selective chemical labeling method (aka m^6^A-SEAL), which is associated with the action that hm^6^A in RNA can be oxidized by the FTO enzyme of m^6^A, is advanced to particularly identify the transcriptome-wide m^6^A [62]. In addition, although cap m^6^Am, a newly identified reversible RNA modification, can be oxidized to cap hm^6^A_m_, m^6^A-SEAL-seq simply oxidizes internal m^6^A to hm^6^A under the present FTO oxidation conditions.

Furthermore, m^6^A-label-seq is a metabolic labeling method to check the mRNA m^6^A transcriptome wide at base resolution. By feeding a methionine analog, Se-allyl-L-selenohomocysteine, the methyl group on the enzyme cofactor S-adenosyl-L-methionine (SAM), can be replaced by allyl cells. In this way, the assumed position of m^6^A-generating adenosine can be substituted by N^6^-allyladenosine (a^6^A) and modified. The advantages of this method are the clustered m^6^A locations and the ability to identify m^6^A modifications of nuclear nascent RNAs [77].

There is no perfect method to detect m^6^A modifications. Therefore, it is extremely necessary to develop more technologies at high levels of single-base and quantitative sequencing.

### The lncRNAs and m^6^A modifications/regulators and diseases

**Fig. 2:**
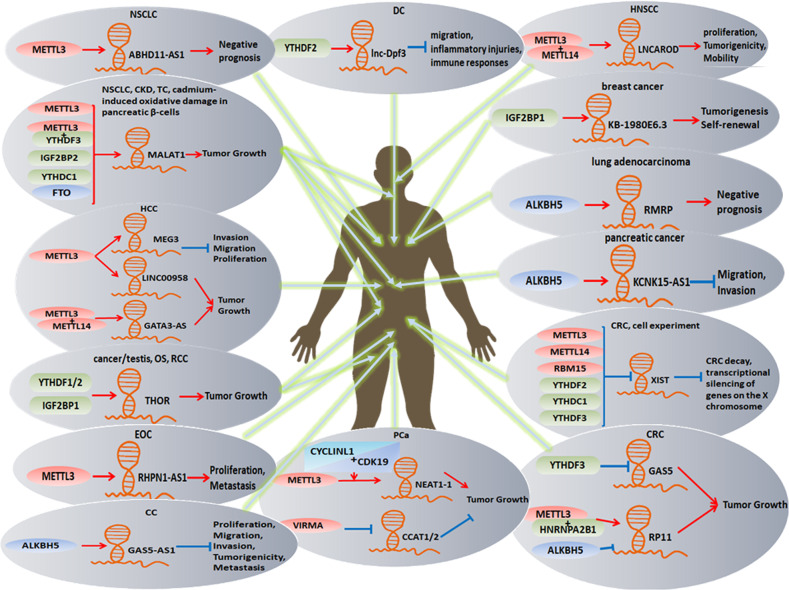
Multiple metabolisms and functions of m^6^A regulators with lncRNAs in the human body. In head and neck squamous cell carcinoma, non-small-cell lung cancer, chronic kidney disease, thyroid cancer, human osteosarcoma, human renal cell carcinoma, epithelial ovarian cancer, breast cancer, lung adenocarcinoma, and cadmium-induced oxidative damage of pancreatic β-cells, the metabolism of m^6^A can accelerate tumor growth, migration, or invasion. However, in dendritic cells, cervical cancer, and pancreatic cancer, the functions of m^6^A regulators trigger the decrease of migration, invasion, or tumorigenicity. However, in the same disease, like hepatocellular carcinoma, colorectal cancer, and prostate cancer, different m^6^A regulators and lncRNAs can, to some extent, display dissimilar functions in the disease process, such as improving tumor growth or decay.

Numerous studies in recent years have elaborated that lncRNAs can be modified by m^6^A and orchestrate m^6^A regulators in disparate tissues or diseases due to the importance of m^6^A modification and the generous acceptance of next-generation sequencing technologies in combination with the escalation of bioinformatics (Fig. 2).

## LncRNA regulation in diseases

### Testis-associated highly conserved oncogenic long non-coding RNA (THOR)

THOR, a singular ultra-conserved cancer-specific lncRNA, mediates cell growth and is entirely expressed in testis and a wide variety of human cancers. In vitro and in vivo, the lack of THOR restrains the proliferation, migration, and invasion of a series of cancer cells [78, 79]. Meanwhile, highly expressed THOR increases IGF2BP1 mRNA expression, which can encourage the survival and proliferation of human osteosarcoma (OS) cells [80]. In addition, the overexpression of THOR also results in the upregulation of IGF2BP1 to enhance the survival and proliferation of human renal cell carcinoma (RCC) cells [81].

### Metastasis-associated lung adenocarcinoma transcript 1 (MALAT1)

It is widely known that MALAT1 with various m^6^A sites is an extraordinary transcript modified by m^6^A with a large percentage of transcripts in several cell lines. As a competitive endogenous RNA, MALAT1 acts as a sponge of miR-1914-3p through Yes-associated protein (YAP) to accelerate the invasion and metastasis of non-small-cell lung cancer (NSCLC) cells [82]. Meanwhile, renal fibrosis plays a crux part in chronic kidney disease (CKD). MALAT1 leads to the promotion of renal fibrosis in patients with obstructive nephropathy (ON). The MALAT1/miR-145/focal adhesion kinase (FAK) pathway has been noted to assume a significant role in TGF-β1-induced renal fibrosis [83]. MALAT1 can aggressively bind competitively to miR-204 to upregulate MYC, thus facilitating the proliferation, migration, and invasion of thyroid cancer (TC) cells and reducing tumor growth and cell apoptosis [84]. In addition, MALAT1 is revealed to participate in cadmium-induced oxidative damage in pancreatic β-cells, which binds to proteins through m^6^A modifications [85].

### Other lncRNAs

The high m^6^A level of nuclear paraspeckle assembly transcript 1 (NEAT1)-1 is correlated with the bone metastasis of prostate cancer (PCa). Ectopically expressed NEAT1-1 can induce cancer cell metastasis to the lung and bone, which is caused by NEAT1-1 with m^6^A site mutations. With the help of METTL3, the new CYCLINL1/CDK19/NEAT1-1 axis can be used as an undeveloped mechanism in the pathogenesis and growth of bone metastatic PCa [86]. CCAT1 and CCAT2, oncogenic lncRNAs, can be adopted as the independent predictors of poor prognosis and reduce the stability through VIRMA downregulation to depress the aggressive phenotypes of PCa cells [87].

In colorectal cancer (CRC) tissues, the direct interactions between growth arrest-specific transcript 5 (GAS5) and the WW domain of YAP facilitate the transferring of endogenous YAP from the nucleus to the cytoplasm and force YAP phosphorylation and afterwards ubiquitin-mediated degradation of YAP, ensuring the obstruction of CRC progression in vitro and vivo [88]. Furthermore, RP11 expression is elevated with increasing stage in CRC patients. RP11 is conclusively correlated with the migration, invasion, and epithelial mesenchyme transition (EMT) of CRC cells in vitro. RP11 can also promote liver metastasis in vivo [28].

LINC00958 is a lipogenesis-related lncRNA whose expression is upregulated in hepatocellular carcinoma (HCC) cell lines and tissues. The high level of LINC00958 can predict the low overall survival of HCC patients. After LINC00958 functions as a sponge of miR-3619-5p, hepatoma-derived growth factor (HDGF) is upregulated, which enhances the lipogenesis, progression, and malignant phenotypes of HCC in vitro and vivo [89]. Compared with adjacent normal tissues, KCNK15-AS1, which can suppress the migration and invasion of MIA PaCa-2 and BxPC-3 cells, reveal low expression in pancreatic cancer tissues [90].

RHPN1-AS1, a sponge of miR-596 to promote LETM1 expression and stimulate the FAK/PI3K/Akt pathway, augments the proliferation and metastasis of epithelial ovarian cancer (EOC) cells in functional experiments in vitro and in vivo [91]. Meanwhile, versus adjacent normal tissues, GAS5-AS1 expression is obviously diminished in cervical cancer (CC) tissues. GAS5-AS1 substantially subdues CC cell proliferation, migration, and invasion in vitro, and specifically weakens CC tumorigenicity and metastasis in vivo. As a result, the low expression of GAS5-AS1 is notably associated with tumor stage, distant and lymphatic metastasis, and poor prognosis in CC patients [92].

LNCAROD, an oncogenic lncRNA, displays differential expression between head and neck squamous cell carcinoma (HNSCC) and normal samples, and the overexpression of LNCAROD is linked to the advanced T stage and poor overall survival of HNSCC. The decline in cell proliferation and mobility in vitro and tumorigenicity in vivo is attributed to the loss of LNCAROD expression, but the result is antithesis when LNCAROD is overexpressed [93].

LncRNA Dpf3 has the capability of manipulating dendritic cell (DC) migration which can be inhibited by CCR7. The DC migration is vital for the stabilization of immune homeostasis and the inauguration of protective immunity. The loss of DC-specific lncRNA Dpf3 can increase the CCR7-mediated DC migration, which contributes to overstating adaptive immune reactions and inflammatory injuries. In the meantime, the binding between lncRNA Dpf3 and HIF-1α can decrease the transcription of the HIF-1α-dependent glycolytic gene Ldha, thereby frustrating DC migratory capacity and glycolytic metabolism [94].

ABHD11-AS1 is highly expressed in NSCLC tissues and cells, and this upregulation in the ectopic area shared a tight association with the negative prognosis of NSCLC patients [95]. Just like ABHD11-AS1, KB-1980E6.3 also shows an association with the negative prognosis of breast cancer through its abnormal overexpression in breast cancer clinical tissues. In vitro and in vivo, the upregulated KB-1980E6.3 can modulate the tumorigenesis and self-renewal of breast cancer stem cells in the hypoxic microenvironment [96]. MEG3 overexpression constrains the proliferation, migration, and invasion of HCC cells. However, the anti-cancer effect of MEG3 can be negated by the high level of miR-544b [97]. Also, cell proliferation, migration, and invasion are reduced and cell apoptosis is increased in RMRP-knocked down lung adenocarcinoma cell lines. The findings indicate that the aberrant upregulation of RMRP is strictly related to the negative prognosis of patients with lung adenocarcinomas [98] (Table 1).

**Table 1:** LncRNA regulation in diseases.

lncRNA	Mechanism	Disease	Function	ref
THOR	Stabilize mRNA	Cancer/testis	Maintain the oncogenic role	[78, 79]
THOR	Upregulate mRNA expression	OS	Increase survival and proliferation	[80]
THOR	Upregulate IGF2BP1	RCC	Increase survival and proliferation	[81]
MALAT1	Sponge miR-1914-3p	NSCLC	Increase invasion and metastasis	[82]
MALAT1	Upregulate	CKD	Increase expression	[83]
MALAT1	Sponge miR-204	TC	Increase proliferation, migration, and invasion of TC cells and weak tumor growth and cell apoptosis	[84]
MALAT1	Sponge protein	Cadmium-induced oxidative damage in pancreatic β-cells	Increase ROS accumulation and MDA content, decrease SOD activities	[85]
NEAT1-1	Upregulate	PCa	Increase bone and lung metastasis	[86]
CCAT1/2	Unstabilize	PCa	Decrease proliferation, migration	[87]
GAS5	Interaction with WW domain	CRC	Degradation of YAP to obstruct CRC progression	[88]
RP11	Upregulate	CRC	Increase migration, invasion, epithelial-mesenchymal transition (EMT), and liver metastasis	[28]
LINC00958	Sponged miR-3619-5p	HCC	Increase lipogenesis, progression, malignant and phenotypes	[89]
KCNK15-AS1	Downregulate	Pancreatic cancer	Increase migration and invasion	[90]
RHPN1-AS1	Sponge miR-596	EOC	Increase proliferation and metastasis	[91]
GAS5-AS1	Downregulate	CC	Increase proliferation, migration, invasion, tumorigenicity, and metastasis	[92]
LNCAROD	Upregulate	HNSCC	Increase proliferation, mobility, and tumorigenicity	[93]
lnc-Dpf3	Downregulate	DC	Overstate adaptive immune responses and inflammatory injuries	[94]
ABHD11-AS1	Upregulate	NSCLC	Negative prognosis	[95]
KB-1980E6.3	Upregulate	Breast cancer	tumorigenesis and self-renewal	[96]
MEG3	Downregulate	HCC	Decrease proliferation, migration, and invasion	[97]
RMRP	Upregulate	lung adenocarcinoma	Negative prognosis	[98]

### The interactions between lncRNAs and m^6^A modifications/regulators

#### THOR

With the high enrichment of m^6^A in THOR transcripts, YTHDF1 and YTHDF2 can mediate the stabilization and degradation of THOR. The interaction of these m^6^A-dependent RNA-proteins can ensure the stability of the oncogenic part of THOR. Meanwhile, the interaction between THOR and IGF2BP1 illustrates that the mRNA activity stability of IGF2BP1 is enhanced by THOR [78, 79]. Furthermore, the target mRNA of IGF2BP1 can also be downregulated through silenced THOR [80]. More importantly, IGF2BP1 expression can also be elevated by the overexpression of THOR, which can facilitate the survival and proliferation of human RCC cells [81].

#### MALAT1

In NSCLC, the high level of m^6^A modifications mediated by METTL3 can augment MALAT1 expression. In the meantime, the METTL3/YTHDF3 complex can stabilize MALAT1 [82]. METTL3 can be upregulated and considered to increase MALAT1 expression in TGF-β1-treated HK2 cells. Thus, m^6^A modifications can afflict MALAT1 expression and possibly exert effects on the MALAT1/miR-145/FAK pathway in renal fibrosis during CKD [83]. MALAT1 and IGF2BP2 are identified to be highly expressed in TC, which is correlated with lowly expressed miR-204. Because IGF2BP2 is confirmed as a target gene of miR-204, MALAT1 can competitively bind to miR-204 to reduce IGF2BP2 expression and elevate MYC expression through m^6^A modifications [84]. In addition, MALAT1, which lacks m^6^A modifications, inhibits the metastatic potential of cancer cells. The concatenated m^6^A residues on MALAT1 can allow YTHDC1 to obtain nuclear speckles for modifying the expression of key oncogenes to enhance the metastatic potential of cancer cells [99]. MALAT1 is determined to decrease with the positive relationship with m^6^A modifications (including METTL3 and FTO), proving that m^6^A modifications and m^6^A-modified lncRNAs potentially are implicated in oxidative damages [85].

#### X-inactive specific transcript (XIST)

XIST is a lncRNA that modulates the transcriptional silencing of genes on the X chromosome. The knockdown of METTL3 or RBM15 can damage the transcriptional silencing of XIST-mediated genes. RBM15 can regulate XIST to tie up the m^6^A-methylation complex and be linked to particular sites in RNA. Moreover, YTHDC1 prefers to detect m^6^A in XIST and maintains the XIST-mediated gene silencing with the loss of m^6^A [100]. XIST can also be documented as a downstream target of METTL14. With the reorganization by YTHDF2, XIST methylated by m^6^A can cause the decay of CRC [101].

#### Other lncRNAs

In CRC, it is worth noting that as a new target of YAP, YTHDF3 can be a linchpin in the YAP pathway which contributes to m^6^A-modified GAS5 degradation. In clinical, the protein levels of YAP and YTHDF3 are negatively related to GAS5 expression in cancer tissues from CRC patients [88]. The overexpression of METTL3 can enhance RP11 expression and the RNA-protein binding between RP11 and HNRNPA2B1. The expression of RP11 can be repressed by ALKBH5 overexpression. These results suggest that m^6^A mediates RP11 expression in CRC cells [28].

In PCa tissues, the m^6^A level of NEAT1-1 is an influential predictor of eventual death. A lack of NEAT1-1 or the decreased m^6^A of NEAT1-1 causes conspicuous damages to Pol II Ser-2p levels in the promoter of RUNX2. CYCLINL1 is not correlated with NEAT1-1 in P-18 cells, which knocks down METTL3. Because of the pivotal role of m^6^A on NEAT1-1 in modifying Pol II ser2 phosphorylation, NEAT1-1 has the potential to be an innovative and specific target for the treatment and diagnosis of bone metastasis cancers [86]. The knockdown of VIRMA can crucially diminish the level of m^6^A, which causes the low expression of CCAT1/2. Furthermore, the repression of VIRMA and m^6^A can reduce the stability and enrichment of CCAT1/2 transcripts. As a result, VIRMA is essential for maintaining the level of m^6^A, and malignant phenotypes can be suppressed by decaying CCAT1/2 transcripts [87].

In HCC, GATA3-AS, which can be transcribed from the antisense strand of the GATA3 gene, acts as a guidance element in the preferential interaction between the METTL3/METT14 complex and the GATA3 pre-mRNA. Ultimately, the growth and metastasis of liver cancer, which are driven by the METTL3/METT14 complex and GATA3-AS, are regulated by GATA3 [102]. LINC00958 can be upregulated by stabilizing its RNA transcript in the presence of METTL3-mediated m^6^A modification in HCC [89]. Meanwhile, ALKBH5, which can m^6^A-demethylate KCNK15-AS1 and mediate cell motility induced by KCNK15-AS1, exhibits low expression, thus considerably augmenting total RNA methylation in pancreatic cancer cells [90].

Following the knockdown of METTL3, the expression and stabilization of RHPN1-AS1 are decreased in EOC, suggesting that m^6^A modifications can improve the transcriptional stability and m^6^A level of RHPN1-AS1, which may explain the results that RHPN1-AS1 is remarkably overexpressed [91]. However, GAS5-AS1 stabilization can be enhanced through the interactions between GAS5-AS1 and ALKBH5 and the depression of GAS5-AS1 m^6^A modifications in CC [92]. More intriguingly, METTL3/14-mediated m^6^A modifications promote the stability of LNCAROD, and the dysregulation of m^6^A may cause the abnormal expression of LNCAROD in HNSCC [93].

In DC, the CCR7 chemokine receptor can accelerate the quick temporary migration to empty lymph nodes. CCR7 stimulation reduces RNA decay by decreasing m^6^A modifications, thus upregulating lncRNA Dpf3. Meanwhile, although CCR7 induction exerts no direct impact on YTHDF2, it can diminish the recognition of YTHDF2 to m^6^A sites of lncRNA Dpf3. In conclusion, CCR7 stimulation elevates lncRNA Dpf3 expression by the m^6^A demethylation and the reduction of m^6^A-related RNA deterioration of lncRNA Dpf3 [94].

The m^6^A modification can be increased by the transfection of METTL3 in NSCLC cells to increase the stability of ABHD11-AS1. Consequently, the overexpression of ABHD11-AS1 augments the effects of Warburg on NSCLC, including excess glucose, lactate production, and ATP accumulation, and the overexpression of ABHD11-AS1 prominently silences the protein level of KLF4 [95]. The KB-1980E6.3/IGF2BP1/c-Myc axis modifies the coding region instability determinant (CRD) mRNA of c-Myc to ensure the stabilization of c-Myc mRNA [96]. In addition, METTL3 silencing reduces the m^6^A modification in MEG3 and conspicuously diminishes the stabilization of MEG3 [97]. ALKBH5 can upregulate RMRP through the demethylation of m^6^A, and the tumorigenesis can be proscribed by ALKBH5 knockdown in vitro and in vivo, representing that ALKBH5 may be directly correlated with RMRP function to modulate lung adenocarcinoma tumor cells [98] (Table 2).

**Table 2:** The interactions between lncRNAs and m^6^A modifications/regulators.

lncRNA	Mechanism	Protein	Disease	ref
THOR	Stabilized by YTHDF1/2	YTHDF1/2	Cancer/testis	[78]
THOR	Stabilize mRNA	IGF2BP1	Cancer/testis	[79]
THOR	Downregulate target mRNA by silence	IGF2BP1	OS	[80]
THOR	Upregulate IGF2BP1	IGF2BP1	RCC	[81]
MALAT1	Upregulate & stabilize	METTL3& METTL3/YTHDF3 complex	NSCLC	[82]
MALAT1	Upregulate	METTL3	CKD	[83]
MALAT1	Competitive	IGF2BP2	TC	[84]
MALAT1	Recruit YTHDC1	YTHDC1		[99]
MALAT1	Positive correlate	METTL3 & FTO	Cadmium-induced oxidative damage in pancreatic β-cells	[85]
XIST	Switch off or on to transcriptional silencing	METTL3, RBM15 or YTHDC1		[100]
XIST	Target or decay	METTL14 or YTHDF2	CRC	[101]
GAS5	Negative correlate	YTHDF3	CRC	[88]
RP11	Upregulate, bind with HNRNPA2B1 or downregulate	METTL3, HNRNPA2B1 or ALKBH5	CRC	[28]
NEAT1-1	Damage Pol II Ser-2p level	METTL3	PCa	[86]
CCAT1/2	Downregulate	VIRMA	PCa	[87]
GATA3-AS	Target induction	METTL3/METT14 complex	HCC	[102]
LINC00958	Stabilize transcript	METTL3	HCC	[89]
KCNK15-AS1	Negative correlate	ALKBH5	Pancreatic cancer	[90]
RHPN1-AS1	Increase stabilize by m^6^A	METTL3	EOC	[91]
GAS5-AS1	Increase stabilize by unm^6^A	ALKBH5	CC	[92]
LNCAROD	Increase stabilize by m^6^A	METTL3/14	HNSCC	[93]
lnc-Dpf3	Increase stabilize by unm^6^A	YTHDF2	DC	[94]
ABHD11-AS1	Increase stabilize by m^6^A	METTL3	NSCLC	[95]
KB-1980E6.3	Increase stabilize by m^6^A	IGF2BP1	Breast cancer	[96]
MEG3	Stabilize by m^6^A	METTL3	HCC	[97]
RMRP	Increase by unm^6^A	ALKBH5	Lung adenocarcinomas	[98]

#### The circRNAs and m^6^A modifications/regulators and diseases

**Fig. 3:**
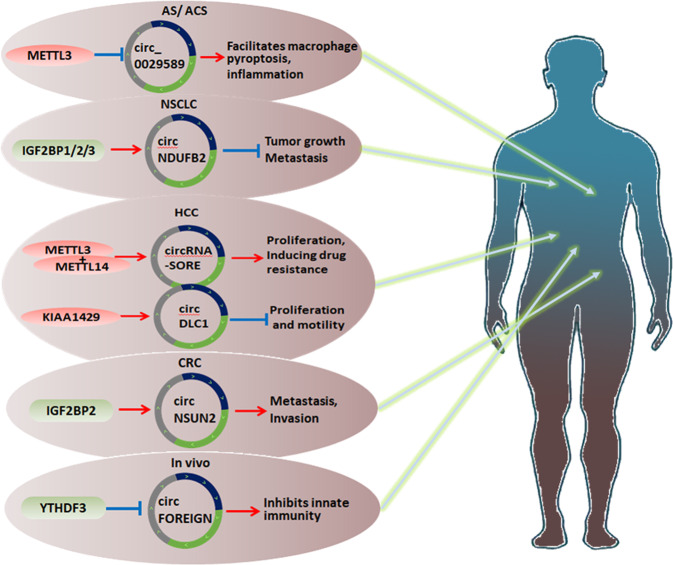
Multiple metabolisms and functions of m^6^A regulators with circRNAs in the human body. In head and neck squamous cell carcinoma, colorectal cancer, non-small-cell lung cancer, atherosclerosis, acute coronary syndrome, and in vivo, m^6^A regulators with circRNAs exert various effects, such as facilitating macrophage pyroptosis, inflammation, metastasis, and invasion.

In contrast to linear RNAs (such as lncRNAs) with a 5′-UTR cap and a 3′-UTR tail, the special circular covalently closed structure of circRNAs can stabilize the architecture against the damages by exonucleases [103]. Moreover, similar to lncRNAs, circRNAs can also be methylated or demethylated by m^6^A modifications. It is widely accepted that circRNAs are critical for the occurrence, development, and prognosis of general diseases, such as cancers and immune disorders. For mechanisms, the functions of circRNAs, including particularly sponging and recruiting miRNAs [104], competitively binding to gene sites, and upregulating or downregulating and transcribing protein or mRNA expression, can mediate essential cellular processes, such as proliferation, differentiation, apoptosis, and metabolism [10, 105] (Fig. 3).

In fact, little is known about the mechanisms of the relations of m^6^A-circRNAs in cancers and diseases. In HCC cells, METTL3 or METTL14 silencing decreases the level of m^6^A, thus inactivating circRNA-SORE. The m^6^A modification enhances the stability of circRNA-SORE, a sponge of miR-103a-2-5p and miR-660-3p to isolate them. After that, the Wnt/β-catenin pathway can be activated competitively, hence inducing the drug fast of sorafenib in HCC cells [106]. CircNSUN2 expression is remarkably higher in tissues of CRC patients with liver metastasis (LM) than in original CRC tissues. Furthermore, the invasion and metastasis of CRC cells can be accelerated by the overexpression of circNSUN2, the m^6^A modification of circNSUN2, and the formation of a circNSUN2/IGF2BP2/HMGA2 RNA-protein ternary complex [107]. For patients with acute coronary syndrome (ACS), the levels of m^6^A-modified circ_0029589 and METTL3 are markedly increased by upregulating IFN regulatory factor-1 (IRF-1), which can cause circ_0029589 inhibition. Thus, macrophage pyroptosis and inflammation can be facilitated by IRF-1 in ACS and atherosclerosis (AS) [108]. CircFOREIGN exhibits the functions of producing antibodies and inducing anti-tumor immunity and antigen-specific T cell activation. However, due to the isolation of m^6^A-circRNA with YTHDF2, m^6^A modifications can restrict innate immunity in vivo [109]. Since circNDUFB2 upregulation can suppress the tumor growth and metastasis of NSCLC cells, circNDUFB2 is downregulated in NSCLC tissues and is inversely related to the malignant characteristics of NSCLC. As a framework, circNDUFB2 can facilitate the interplay between TRIM25 and IGF2BP1/2/3, which demonstrates a vigorous influence on tumor progression and metastasis [110]. CircDLC1 is negatively manipulated by KIAA1429, which shows a low level in HCC tissues and is associated with an obviously favorable prognosis [111] (Table 3).

**Table 3:** The interactions between circRNAs and m^6^A modifications/regulators in diseases.

circRNA	Mechanism	Disease	m^6^A Protein	Function	ref
circRNA-SORE	Sponge miR-103a-2-5p & miR-660-3p	HCC	METTL3/14	Increase stabilize by m^6^A and competitively activating the Wnt/β-catenin pathway and inducing sorafenib resistance	[106]
circNSUN2	Upregulate	CRC	IGF2BP2	Stabilize HMGA2 by circNSUN2/IGF2BP2/HMGA2 RNA-protein ternary complex and promotes LM in PDX metastasis models in vivo and accelerates cancer cells invasion in vitro	[107]
circ_0029589	Downregulate by IRF-1	AS/ ACS	METTL3	Downregulate by up methylation and facilitates macrophage pyroptosis and inflammation	[108]
circFOREIGN	Sequester m^6^A-circRNA	In vivo	YTHDF2	Sequester m^6^A-circRNA and inhibits innate immunity	[109]
circNDUFB2	Downregulate	NSCLC	IGF2BP1/2/3	Increase the interplay and inhibits tumor growth and metastasis	[110]
circDLC1	Downregulate	HCC	KIAA1429	Negatively correlates to and inhibits cell proliferation and motility	[111]

## Conclusion and further vision

In recent years, epigenetic modifications of RNA molecules have increased contemplation with an avalanche of publicity. As multiple key bridges of biological actions, RNA mediates the architecture of whole chromosomes and the functions of transcription, translation, enhancement, and inhibition. Compared with short RNAs, such as miRNAs, lncRNAs are widely regarded as RNA polymerase II (Pol II)-transcribed molecules with different types and dimensions >200 nt. Accumulating studies unveil that lncRNAs have a series of functions of mediating transcriptional and post-transcriptional gene expression in a wide range of mechanisms and diseases [5]. Meanwhile, the majority of circRNAs are ncRNAs, some of which in the cytoplasm can show the potential capability of peptide translation [11].

In a large number of primary and transformed cells, the dynamic reversibility of m^6^A can occur in profuse locations of mRNAs, lncRNAs, and circRNAs, even though m^6^A is not directly linked to the progression of tumors or diseases [18]. With m^6^A-seq, MeRIP-Seq, SCARLET, and innovatively developed detection methods, such as m^6^A-SEAL and m^6^A-label-seq, m^6^A-methylated RNA pieces are always recognized and m^6^A is noticeably expressed in the 3′-UTR near mRNA stop-codons and long internal exons in different cell/tissue special sites [24]. Thus, biological actions result in the particular transcriptome topology of m^6^A. With the supplementary modification of m^6^A, the functions of lncRNAs and circRNAs encompass an abundant diversity of significant biological phenomena, including the imprinting of genomes [4], the sponging or stabilization of miRNAs or proteins, and the upregulation or downregulation of related genes. Furthermore, ncRNAs can feedback to afflict the effect of m^6^A modification, then complicating the outcomes of pathophysiological activities in vitro and in vivo.

Regardless of how many DNAs, RNAs, proteins, and their complexes are involved, there still are only four steps required from genome to proteome: RNA production, RNA degradation, protein production, and protein degradation. Unfortunately, because of the limits of methods and other factors, we still only reveal the tip of the iceberg in the physiological and pathological activities of cells and organisms. There are a bunch of ncRNAs whose functions and interactions modulated by m^6^A modifications need more characterization studies and detections to be recognized, which may be the focus of future studies. In summary, with the deeper investigations into lncRNAs, circRNAs, and m^6^A, there may be some novel therapies, effective strategies, or sensitive detection methods to cure or screen for cancers, especially in combination with immunotherapies.
